# Longitudinal study of body mass index in relation to Alzheimer's disease pathology and symptomatology in Down syndrome

**DOI:** 10.1002/alz.70387

**Published:** 2025-06-22

**Authors:** Victoria L. Fleming, Brian C. Helsel, Lauren T. Ptomey, Benjamin L. Handen, Sharon J. Krinsky‐McHale, Christy L. Hom, Matthew Zammit, Davneet Minhas, Weiquan Luo, Charles Laymon, Joseph H. Lee, Ira Lott, Annie Cohen, Beau M. Ances, Adam M. Brickman, Margaret Pulsifer, Isabel C. H. Clare, H. Diana Rosa, Florencia Lai, Jordan Harp, Fredrick Schmitt, Julie Price, Shahid H. Zaman, Elizabeth Head, Mark Mapstone, Bradley T. Christian, Ozioma Okonkwo, Sigan L. Hartley, Alzheimer's Biomarkers Consortium–Down Syndrome

**Affiliations:** ^1^ Waisman Center University of Wisconsin—Madison Madison Wisconsin USA; ^2^ Department of Human Development and Family Studies University of Wisconsin—Madison Madison Wisconsin USA; ^3^ Department of Neurology University of Kansas Medical Center Kansas City Kansas USA; ^4^ Department of Internal Medicine University of Kansas Medical Center Kansas City Kansas USA; ^5^ Department of Psychiatry University of Pittsburgh Pittsburgh Pennsylvania USA; ^6^ Department of Psychology New York Institute for Basic Research in Developmental Disabilities New York New York USA; ^7^ Deparment of Psychiatry & Human Behavior University of California, Irvine Irvine California USA; ^8^ Department of Radiology University of Pittsburgh Pittsburgh Pennsylvania USA; ^9^ Department of Bioengineering University of Pittsburgh Pittsburgh Pennsylvania USA; ^10^ Taub Institute for Research on Alzheimer's Disease and the Aging Brain Sergievsky Center and Department of Neurology Vagelos College of Physicians and Surgeons Columbia University New York New York USA; ^11^ Department of Pediatrics University of California Irvine School of Medicine Irvine California USA; ^12^ Behavioral Health Service Line VA Pittsburgh Healthcare System Pittsburgh Pennsylvania USA; ^13^ Department of Neurology Washington University School of Medicine in St. Louis St. Louis Missouri USA; ^14^ Department of Psychiatry Massachusetts General Hospital Harvard Medical School Boston Massachusetts USA; ^15^ Department of Psychiatry University of Cambridge Cambridge UK; ^16^ Department of Neurology University of Kentucky Lexington Kentucky USA; ^17^ Department of Radiology Massachusetts General Hospital Harvard Medical School Boston Massachusetts USA; ^18^ Department of Pathology & Laboratory Medicine University of California Irvine School of Medicine Irvine California USA; ^19^ Department of Neurology University of California Irvine School of Medicine Irvine California USA; ^20^ Department of Psychiatry University of Wisconsin—Madison Madison Wisconsin USA; ^21^ Department of Medical Physics School of Medicine and Public Health University of Wisconsin‐Madison Madison Wisconsin USA; ^22^ Department of Medicine University of Wisconsin—Madison Madison Wisconsin USA

**Keywords:** ABC‐DS, Alzheimer's disease, amyloid beta, cognition, Down syndrome, trisomy 21, weight loss

## Abstract

**INTRODUCTION:**

Weight loss has been linked to early Alzheimer's disease (AD) pathology, possibly through metabolic dysregulation. We examined changes in body mass index (BMI) in relation to AD biomarkers (amyloid beta [Aβ] and tau) and cognitive decline in adults with Down syndrome (DS). We hypothesized that BMI decline would track with early AD pathology and cognitive decline.

**METHODS:**

Adults with DS (*N* = 467; *M_age _* = 43.67 ± 10.06) completed one to four data cycles (≈16 months apart). Linear mixed models examined BMI change over time by age, positron emission tomography (PET) Aβ and tau, and changes in memory and dementia symptoms.

**RESULTS:**

BMI declined with age‐by‐time (*β* = −0.014, *p* = 0.002) and baseline PET Aβ‐by‐time (*β* = −0.005, *p* = 0.002). On average, BMI decline began in the early 40s and was related to decline in memory and overall cognitive functioning.

**DISCUSSION:**

Weight loss is associated with the presence of Aβ and cognitive decline in adults with DS. Longitudinal studies need to clarify directionality and biological mechanisms.

**Highlights:**

- Adults with Down syndrome (DS) are at an elevated risk for Down Syndrome assocaited Alzheimer's disease (DSAD).
- On average, adults with DS experience body mass index (BMI) decline beginning in their early 40s.
- Positron emission tomography amyloid beta deposition is associated with greater decline in BMI in adults with DS.
- Across time, AD‐related memory declines are associated with BMI decline.
- BMI decline should be part of DSAD screening tools, as it is an important part of DSAD clinical disease expression.

## 1 BACKGROUND

Weight loss is linked to preclinical stage of Alzheimer's disease (AD), when early pathological changes are present but cognition remains intact, in the general population.^1^, ^2^ In studies of late‐onset AD (LOAD), weight loss is documented in the years immediately preceding AD dementia onset.^3^, ^4^, ^5^ For example, Buchman and others^3^ found that a body mass index (BMI) decrease of 1 kg/m^2^ per year (vs no change) was associated with a 35% greater risk of developing AD within 5 years. Weight loss is associated with elevated cerebrospinal fluid (CSF) and positron emission tomography (PET) biomarkers of amyloid beta (Aβ) plaques and neurofibrillary tau tangles,^6^, ^7^ two hallmark pathologic features of AD.^8^, ^9^ However, the biological mechanisms linking weight loss and AD remain unclear. One possibility is that Aβ accumulation and/or downstream neurodegeneration may disrupt hormone regulation, including hormones like leptin^10^ and adiponectin.^11^ Indeed, there are altered plasma and CSF adiponectin levels in individuals with mild cognitive impairment (MCI) and AD, suggesting metabolic dysfunction.^11^ Neurodegeneration in brain regions related to memory and sensory processing may also impair weight regulation and appetite control.^12^, ^13^ As such, unintentional weight loss can serve as a meaningful clinical sign of impending AD dementia in the general population. It is unclear, however, how weight loss may be related to the development of AD in adults with Down syndrome (DS), a group genetically at‐risk for AD (referred to as Down Syndrome assocaited Alzheimer's disease [DSAD]).

Adults with DS, or trisomy 21, have a 90% lifetime risk for developing DSAD due to the triplication of the amyloid precursor protein (*APP*) gene on chromosome 21.^14^ Having three copies of the *APP* gene results in a 1.5× greater production of Aβ across the lifespan.^15^, ^16^ In DS, brain Aβ deposition is evident in the 30s^17^, ^18^ and is followed by intracellular neurofibrillary tau tangles in the 40s and 50s.^14^, ^19^, ^20^ The median age of symptomatic DSAD is 53 years.^21^ It is estimated that 60 to 90% of adults with DS are overweight or obese.^22^, ^23^, ^24^ Furthermore, BMI is at its highest during the third decade of life.^25^, ^26^ In a cross‐sectional study, Agiovlasitis and others^25^ found that the BMI of adults with DS is likely to increase through the 30s, with significant decrease in BMI thereafter. Only a handful of studies examined the association between weight loss and DSAD.^27^, ^28^, ^29^ Bayen and others^27^ reported that adults with DS and AD were twice as likely to lose weight as adults with DS who were cognitively stable. In a large cross‐sectional study of adults with DS, using age‐trajectory estimates, BMI decreased by ≈0.23 kg/m^2^ per year starting in the late 30s, which aligns with Aβ deposition.^28^

The current study builds on these prior studies to longitudinally assess the association between baseline PET Aβ and tau burden, change in memory and dementia symptoms (e.g., mental status), and change in BMI across time. Analyses drew on the longitudinal cohort study of adults with DS enrolled in the Alzheimer's Biomarkers Consortium–Down Syndrome (ABC‐DS^30^). Analyses leveraged up to four time points (follow‐up to ≈7 years) of data collection. The study aims included: (1) evaluate change in BMI across time and by age in adults with DS; (2) determine the effect of baseline PET Aβ and tau on BMI change; and (3) evaluate the association between change in BMI and change in memory and dementia symptoms. Based on prior cross‐sectional research,^25^, ^28^ BMI was hypothesized to increase or remain stable through the late 30s and then decline after age 40 years. Based on research on LOAD,^6^, ^7^ higher baseline PET Aβ and tau were expected to be associated with decreases in BMI overtime. Declines in BMI were expected to be associated with declines in memory and increases in dementia symptoms across time. Moreover, the above associations between BMI decline and AD pathology and cognitive decline were expected to be observed even in adults with DS who did not have clinical dementia, and thus occur early on in the unfolding of DSAD. Understanding of the connection between weight loss and DSAD has important implications for AD screening and for managing the clinical expression of DSAD.

## 2 METHODS

### 2.1 Participants

Analyses included 467 adults with DS enrolled in one of nine ABC‐DS, a large multi‐site longitudinal study focused on identifying early biomarkers of AD in DS.^30^ Inclusion criteria at baseline included age ≥25 years, “mental age” ≥30 months, and chromosomal analysis confirming trisomy 21; exclusion criteria included contraindications for brain imaging or the presence of any untreated medical or mental health conditions that could impact cognitive functioning.

### 2.2 Procedures

Participants completed a multi‐day study visit that involved a battery of directly administered cognitive measures, blood draw, neurophysical exam that included assessment of height and weight, and magnetic resonance imaging (MRI) and PET scans. A study partner attended the visit and reported on the participant's sociodemographics, medical history, activities of daily living, and screens for symptoms of cognitive decline or dementia. Visits were repeated every 16 months (±3 months). Analyses include up to four data collection cycles.

**RESEARCH IN CONTEXT**

1. **Systematic review**: The terms weight, body mass index (BMI), Alzheimer's disease (AD), and Down syndrome (DS) were used to search traditional research databases (i.e., PubMed, Google Scholar). Literature investigating the association between weight and AD in the general adult population and in the DS population were reviewed. There was limited research on weight change and AD in DS that was cross‐sectional in nature. However, the existing literature consistently linked weight loss to AD in the general adult population.
2. **Interpretation**: Adults with DS decline in BMI across adulthood, with the onset of this decline aligning in time with the presence of early AD pathology (i.e., positron emission tomography amyloid positivity) and associated with decline in memory and increased dementia symptoms across time.
3. **Future directions**: Further longitudinal studies are needed to understand mechanisms linking weight loss to DSAD pathology.

### 2.3 Measures

#### 2.3.1 Sociodemographics

The participant's age was reported by the study partner at baseline. The study partner also reported biological sex at birth (male = 1; female = 2) and race/ethnicity (1 = White, 2 = Black; 3 = American Indian; 4 = Asian; 5 = Hispanic). Premorbid intellectual disability (ID) level was based on standardized intelligence quotient (IQ) scores recorded in medical records or obtained in ABC‐DS using the Stanford‐Binet Intelligence Scales, Fifth Edition^31^ abbreviated IQ and/or the Kaufman Brief Intelligence Test, Second Edition^32^ prior to any concerns about cognitive decline. Premorbid ID was coded as: mild (“mental age”: 9 to 14 years; coded = 1), moderate (“mental” age: 4 to 8 years; coded = 2), or severe/profound (“mental age”: <4 years; coded = 3). Genotyping was conducted to determine the presence of apolipoprotein E (*APOE*) ε4 carrier status. Time between data collection cycles in years from baseline was calculated.

#### 2.3.2 BMI

At baseline, height was measured using a tape measure or stadiometer, whereas at each study visit, weight was measured using either a digital or mechanical scale. Both were assessed with shoes off for better accuracy of height and weight and using the same equipment at each visit. BMI was calculated as weight in kilograms divided by height in meters squared. Three categories were defined: Normal + Underweight (<25 kg/m^2^); Overweight (25 to <30 kg/m^2^); and Obese (≥30 kg/m^2^).^33^

#### 2.3.3 Clinical AD status

Clinical AD dementia status was based on a case review consensus process that involved expert DS clinicians, study coordinators, and highly trained and experienced staff who were familiar with each participant. Consensus teams were blind to imaging, ‐omics, and genetic data. All available informant‐reported and direct measures of cognition, adaptive functioning, and behavior, along with medical histories, premorbid ID, neurophysical exam findings, and clinical lab results (i.e., complete blood count and metabolic comprehensive panel.) were reviewed. Using this information, participants were given a status of cognitively stable (defined as no evidence of cognitive or functional decline beyond normal aging; coded = 0), MCI (defined as subtle and/or limited decline in cognition and/or adaptive behavior; coded = 1), AD dementia (defined as significant and sustained declines in cognition or functioning; coded = 2), or unable to determine (meaning that changes in cognition and/or functioning were observed but could have been caused by significant life events or medical history changes = 3).

#### 2.3.4 Cognitive functioning

The modified Cued Recall Test (mCRT)^34^ was used to assess memory. It involves requesting that participants learn and then freely recall a list of 12 pictures. Following three free recall trials, there were cued trials in which a category prompt (e.g., “piece of fruit” for pictures of grapes) was provided verbally. The mCRT Total score is the sum of free and cued recall across three trials, with a higher mCRT Total score indicating better memory performance. The mCRT Total score has been shown to differentiate adults with DS who are cognitively stable from those with MCI or dementia,^35^ and is associated with early Aβ accumulation in DSAD.^36^, ^37^

The Down Syndrome Mental Status Examination (DSMSE)^38^ assesses mental status with items involving recall of personal information, orientation to the day and season, immediate and delayed recall, language, and visuospatial function and praxis. The DSMSE can also distinguish adults with DS with MCI or dementia from those who are cognitively stable.^37^

Finally, the National Task Group‐Early Detection Screen for Dementia (NTG‐EDSD)^39^ is an established informant‐reported measure of symptoms of dementia. The 6‐domain total score ranges from 0 to 51, with higher scores indicating more dementia symptoms and is sensitive to MCI and dementia in DS.^40^

#### 2.3.5 AD pathology

MR scans were acquired on a 3T GE Discovery MR750, Siemens Trio, Siemens Prisma, or GE Signa PET/MR depending on the imaging site in a subsample of participants (*n* = 245). Aβ was assessed using a tracer: [^11^C]‐Pittsburgh compound B ([^11^C] PiB) or florbetapir (AV‐45), whereas tau was assessed using the tracer 18F‐flortaucipir ([^18^F] AV‐1451). Images were taken 50–70 min post‐injection for [^11^C] PiB and 80–100 min for [^18^F] AV‐1451.  Data were reconstructed using iterative methods and corrected for movement, deadtime, and radioactive decay. Images were captured in 5‐min frames and corrected for motion on a frame‐by‐frame basis. High‐resolution T1‐weighted images were acquired using a three‐dimensional (3D) fast spoiled gradient echo (FSPGR) or magnetization prepared rapid acquisition gradient echo (MPRAGE) sequence and used for anatomic reference in PET processing. Through the use of FreeSurfer 5.3, T1‐weighted images were segmented into regions defined by Desikan–Killiany atlas regions.^41^ Results were inspected and 12 high‐quality parcellations were selected as templates. These templates were warped into each participant's native MR space using the Advanced Neuroimaging Tools (ANTs) software package, with a final native space atlas being created by determining the maximum overlap of each parceled region from the 12 templates. Results were accepted or rejected on a visual rating of the final atlas’ adherence to the participant's MR anatomy.

The Centiloid method^42^ was used to calculate amyloid burden from amyloid PET scans. An advantage to the method is that it provides a standardized scale for the two tracers. Briefly, PET scans were registered to the corresponding T1 MRI. The MRI was then warped to the Montreal Neurological Institute (MNI152) template using Statistical Parametric Mapping, version 8, (SPM8). The wrap parameters were then used to cowarp the MRI and PET images. Radioactivity concentration was extracted for the Centiloid standard global region and whole cerebellum using regions of interest (ROIs) predefined on the MNI152 template.^43^ Global SUVr was taken to be the ratio of tracer concentration in the global region to that of the cerebellum. Using linear+constant transformations specified separately for [^11^C]PiB^37^ and [^18^F] AV‐45,^43^ tissue ratios were converted to centiloid values.

For tau, scans were registered to T1 MRI using PMOD software. Through the use of FreeSurfer 5.3, images were segmented into regions defined by Desikan–Killiany atlas regions.^41^ Reported SUVrs were calculated by normalizing concentrations to the cerebellar cortex concentration.

### 2.4 Data analysis

Variable distributions and potential outliers were examined using descriptive statistics, box plots, and histograms. Analysis of variance (ANOVA) and chi‐square tests were run to assess differences in sociodemographic data by BMI categories. Mixed linear models were conducted using the lmer function from the lme4 R package.^44^ Time, age, and their interaction, as well as biological sex, premorbid ID, and *APOE* ε4 status, were included as fixed effects in the models with random intercepts varying among participants and the data collection site. Models were originally conducted including the full sample but were re‐run to include only participants deemed to be cognitively stable. The significance level was set to *p* ≤ 0.05 for the data analysis.

## 3 RESULTS

### 3.1 Preliminary analyses

At baseline, average participant age was 43.67 years (SD = 10.06), ranging from 25 to 81 and having a normal distribution (skewness: 0.14). The average time between study visits was 15.12 (SD = 8.04) months. The average baseline BMI of the participants was 31.45 (SD = 7.34), with 17.13% of participants being underweight or having normal BMI (BMI <25 kg/m^2^), 30.84% being overweight (BMI 25 to <30 kg/m^2^), and 52.04% being obese (BMI ≥30 kg/m^2^). About half of participants were female (46.25%), with a majority were of White, non‐Hispanic (91.01%) race/ethnicity, and 23.98% were *APOE* ε4 carriers. The majority of participants had mild (45.61%) or moderate premorbid ID levels (45.82%), but a subset (8.14%) had severe/profound ID. The majority of participants (73.23%) were cognitively stable at baseline, whereas 57 (12.21%) were deemed to have MCI and 49 (10.49%) had a clinical AD status of dementia. Table 1 provides information about participant characteristics and provides the means and SDs for the main study variables.

**TABLE 1:** Participant characteristics at baseline and mean and SD for the study variables.

	Overall *N* = 467	Normal + Underweight *N* = 80	Overweight *N* = 144	Obese *N* = 243	*p*‐value
Age, M ± SD	43.67 ± 10.06	47.5 ± 10.5	44.3 ± 10.5	42.1 ± 9.2	< 0.001
BMI, M ± SD	31.46 ± 7.34	22.6 ± 1.9	27.4 ± 1.4	36.8 ± 6.1	
**Biological sex, no. (%)**					0.034
Female	216 (46.25%)	30 (37.50%)	60 (41.67%)	126 (51.85%)	
Male	251 (54.75%)	50 (62.50%)	84 (58.33%)	117 (48.15%)	
**Race/ethnicity, no. (%)**					0.385
White, non‐Hispanic	425 (91.01%)	71 (88.75%)	127 (88.19%)	227 (93.42%)	
Black	7 (1.50%)	2 (2.5%)	2 (1.39%)	3 (1.24%)	
American Indian	1 (0.21%)	0 (0.0%)	0 (0%)	1 (0.41%)	
Asian	6 (1.28%)	2 (2.5%)	4 (2.78%)	0 (0.0%)	
Hispanic	23 (4.93%)	4 (5.0%)	10 (6.94%)	9 (3.70%)	
**Diagnosis, no. (%)**					< 0.001
No MCI or dementia	342 (73.23%)	44 (55.0%)	105 (72.92%)	193 (79.42%)	
MCI	57 (12.21%)	20 (25.0%)	22 (15.28%)	15 (6.17%)	
AD dementia	49 (10.49%)	12 (15.0%)	11 (7.64%)	26 (10.70%)	
Unable to determine	19 (4.07%)	4 (5.0%)	6 (4.17%)	9 (3.70%)	
**Premorbid ID level, no. (%)**					0.113
Mild	213 (45.61%)	33 (41.25%)	59 (40.97%)	121 (49.79%)	
Moderate	214 (45.82%)	37 (46.25%)	76 (52.78%)	101 (41.56%)	
Severe/profound	38 (8.14%)	10 (12.50%)	8 (5.56%)	20 (8.23%)	
Unknown	2 (0.43%)	0 (0.0%)	1 (0.69%)	1 (0.41%)	
***APOE* ε4, no. (%)**					0.556
Present	112 (23.98%)	23 (28.75%)	35 (24.31%)	54 (22.22%)	
Absent	340 (72.81%)	53 (66.25%)	104 (72.22%)	183 (75.31%)	
Unknown	15 (3.21%)	4 (5.0%)	5 (3.47%)	6 (2.47%)	

*Note*. Intellectual disability levels reflect the following “mental age”: mild: ≥9 years, moderate: 4 to 8 years; severe/profound: <4 years. Analysis of variance (ANOVA) and Tukey post hoc tests showed significant differences in age by BMI category, with overweight and obese participants being younger than those in the underweight/normal group (*F*(2, 464) = 9.62, *p* < 0.001). Females were more likely than males to be overweight or obese (*χ* ^2^(2, 467) = 6.75, *p* = 0.034). Cognitively stable individuals were more likely to be in the normal or overweight BMI groups compared to those with MCI or AD dementia (*χ* ^2^(6, 467) = 26.92, *p* < 0.001). No significant differences were found in BMI category by race, premorbid ID, or *APOE* ε4 status.

Abbreviations: *APOE* ε4, apoliproprotein E4; AD, Alzheimer's disease; BMI, body mass index; ID, Intellectual Disability; MCI, mild cognitive impairment.

Analyses included participants with at least one time point (*n* = 467) of BMI data. The majority of participants provided multiple timepoints: two (*n* = 177, 37.90%), three (*n* = 144, 30.84%), and four (*n* = 128, 27.41%). An ANOVA and follow‐up Tukey tests indicated that participants with only one time point of BMI data were younger than those with multiple time points of data (*F*(4, 464) = 18.789, *p *< 0.001). There were no statistically significant differences between participants with one versus multiple time points of data in BMI (*F*(4, 464) = 1.347, *p *= 0.251), biological sex (*χ* ^2^[4,465] = 2.007, *p *= 0.735), race *χ* ^2^[4,465] = 4.922, *p *= 1.00), or premorbid ID (*χ* ^2^[4,465] = 4.080, *p *= 0.982).

Of the 467 participants, PET Aβ was available for 245 (52.46%) and tau PET was available for 134 (28.69%). Neuroimaging data were either not collected or not yet processed in a harmonized way for the remaining participants. Analyses compared the sociodemographics of participants with versus without neuroimaging data. Compared with those for whom neuroimaging data was not available, the participants with neuroimaging data were younger (*t*(435) = 6.792, *p *< 0.001) but did not differ in biological sex (*χ* ^2^[1,465] = 1.024, *p *= 0.311), *APOE* ε4 status (*χ* ^2^[1,465] = 2.06, *p *= 0.357), BMI (*χ* ^2^[1,465] = −2.04, *p *= 0.061), race (*χ* ^2^[1,465] = 2.018, *p *= 0.569), or premorbid ID level (*χ* ^2^[1,465] = 1.394, *p *= 0.498). The participants with imaging data also scored higher on the mCRT (*t*(422) = −2.173, *p *< 0.001) and DSMSE (*t*(460) = −3.730, *p *< 0.001), but did not differ on the NTG (*t*(466) = 1.385, *p *= 0.167). This pattern likely reflects recruitment practices, as sites focused on imaging recruited through legacy studies that involved younger participants than the sites not focused on imaging. However, it is also likely that younger adults with DS and those with fewer co‐occurring medical conditions may be more willing or able to undergo neuroimaging protocols.

The results of an ANOVA and follow‐up Tukey tests indicated there was a statistically significant difference in age among BMI categories (underweight/normal weight, overweight, and obese) (*F*(2, 464) = 9.615, *p *< 0.001). Participants who were obese or overweight were younger than those with BMI in the normal or underweight categories (Table 1). Pearson chi‐square statistics indicated that female participants were more likely to be in the overweight and obese group compared to male participants (*χ* ^2^[2,467] = 6.75, *p *= 0.034). Participants who were cognitively stable were more likely to be in the normal or overweight groups than were participants with MCI or dementia (*χ* ^2^[6,467] = 26.92, *p *< 0.001). There was no statistically significant differences between BMI category by race (*χ* ^2^[10,467] = 10.65, *p *= 0.385), premorbid ID (*χ* ^2^[4,465] = 7.48, *p *= 0.113), or *APOE* ε4 status (*χ* ^2^[4,467] = 3.01, *p *= 0.556).

### 3.2 Change in BMI over time

**FIGURE 1:**
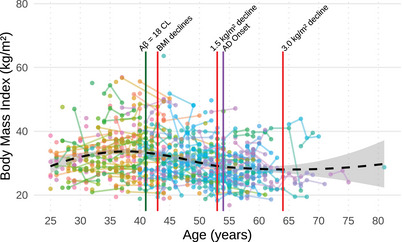
Association between age and body mass index. A LOESS regression was used to model the association between age and BMI (*n* = 467), capturing the non‐linear trend across the age span. Individual lines are color‐coded to distinguish participants and aid in visualizing individual BMI trajectories across the sample. The green line is the age when Aβ (CL = 18) reaches positivity (age = 41 years). The first red line indicates when BMI starts to significantly decline (age = 43 years), the second red line indicates a 1.5 unit decline (age = 53), and the third red line indicates a 3.0 unit decline (age = 64). The purple line indicates the average age at AD onset (age = 53.98). AD, Alzheimer's disease; Aβ, amyloid beta; BMI, body mass index; CL, Centiloid; kg, kilograms; m, meters.

Table 2 presents results from the linear mixed model analyzing change in BMI across time and time by age, with sociodemographic controls also entered into the model. Premorbid ID and *APOE* ε4 status were not significantly associated with BMI (*β* = −0.861, *p *= 0.092 and *β* = 0.0311, *p *= 0.964). Female participants tended to have a higher BMI than male participants across time (*β* = 2.07, *p *= 0.002). There was a significant positive association between time and change in BMI (*β* = 0.532, *p *= 0.01), indicating an overall increase in BMI over time. Indicated across the entire sample, there was an increase in BMI over time. However, the model shows the effects of time, time by age, with sociodemographic controls effects on change in BMI across time (*β* = −0.011, *p *= 0.002) and time × age (*β* = −0.015, *p *= 0.002) were both significantly negative. Therefore, on average younger individuals gained weight over time, whereas older participants lost weight over time. Model estimates indicate that, on average, weight loss begins at age 43 years, and by age 53 years, an adult with DS would on average experience a 1.5 kg/m^2^ decrease in BMI (from BMI in their early 40s). By age 64 years, adults with DS on average experience a 3 kg/m^2^ decrease in BMI (from BMI prior in their early 40s). Figure 1 displays BMI change over time by age.

**TABLE 2:** Multilevel models of baseline sociodemographics predicting body mass index.

	Estimates				
	Fixed effects	*β*	*SE* β	*t*	*p*‐value
**BMI**	Intercept	34.470	2.056	16.77	<0.001^***^
	Time	0.532	0.208	2.56	0.011^*^
	Age	−0.107	0.034	−3.11	0.002^**^
	Biological sex	2.071	0.647	3.20	0.002^**^
	Premorbid intellectual disability	−0.861	0.510	−1.689	0.092
	*APOE* ε4	0.031	0.680	0.046	0.964
	Time × age	−0.015	0.005	−3.201	0.002^**^

*Note*. *N *= 473. Unstandardized coefficients (*β*s) are presented. All models include a random intercept and include site as a random intercept.

Abbreviations: *APOE*, apolipoprotein E; BMI, body mass index.

* *p* < 0.05.

** *p* < 0.01.

*** *p* < 0.001.

The model was re‐run only including the 342 participants who were cognitively stable at baseline. Significant associations remained; time (*β* = 0.468, *p *= 0.034) and time × age (*β* = −0.013, *p *= 0.012) predicted decline in BMI over time. To further to explore the impact of weight changes, the model was re‐run excluding participants who experienced a BMI changes of ≥10 (*n* = 10). The results remained consistent: both age (*β* = −0.115, *p *= 0.001) and time × age (*β* = −0.008, *p *= 0.042) remained statistically significant.

### 3.3 BMI and baseline PET Aβ and tau

Table 3 presents linear mixed models of the effect of baseline PET Aβ (Model 1) and also baseline PET Aβ and tau (Model 2) on BMI, with time, time × age, and sociodemographic controls also included. Interactions of time × PET Aβ and time × PET tau were also included as predictors. Biological sex had a significant positive effect on BMI (*β* = 2.787, *p *= 0.002); as in earlier models, female participants had a higher average BMI than male participants. There was also a significant effect of time × PET Aβ on BMI (*β* = −0.005, *p *= 0.003), even when adjusting for age, such that higher baseline PET Aβ predicted greater BMI decreases across time. This finding suggests that PET Aβ contributes to BMI decline above and beyond age‐related effects.

**TABLE 3 alz70387-tbl-0003:** Linear mixed modeling for baseline Alzheimer's disease pathology and change in body mass index.

		Estimates			
	Fixed effects	*β*	SE β	*t*	*p*‐value
Model 1	Intercept	31.630	3.345	9.454	< 0.001^***^
	Time	0.029	0.074	0.390	0.697
	Age	−0.080	0.068	−1.173	0.242
	Biological sex	2.787	0.089	2.125	0.002^**^
	Premorbid intellectual disability	−0.453	0.680	−0.667	0.506
	*APOE* ε4	0.857	0.990	0.858	0.391
	Amyloid beta (Aβ)	0.0005	0.017	0.032	0.974
	Time × Aβ	−0.005	0.001	−3.058	0.003^**^
Model 2	Intercept	0.302	7.811	3.860	< 0.001^***^
	Time	−0.537	0.728	−0.737	0.463
	Age	−0.042	−0.120	−0.393	0.695
	Biological sex	3.884	1.315	2.954	0.003^**^
	Premorbid intellectual disability	−0.716	0.982	−0.729	0.468
	*APOE* ε4	−0.274	1.533	−0.179	0.858
	Aβ	0.000	0.052	0.009	0.993
	Tau	−0.416	4.934	−0.084	0.933
	Time × Aβ	−0.013	0.005	−2.304	0.023^*^
	Time × tau	0.056	0.666	0.001	0.405

*Note*: *N *= 245. Unstandardized coefficients (*β*s) are presented. All models include a random intercept and site was included as a random intercept. Model 1 is an Aβ only model and Model 2 consists of Aβ and tau PET.

Abbreviations: *APOE*, apolipoprotein E; BMI, body mass index.

^*^ *p* < 0.05.

** *p* < 0.01.

*** *p* < 0.001.

**FIGURE 2 alz70387-fig-0002:**
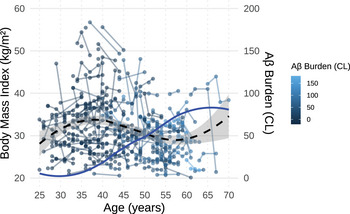
Association between age, amyloid‐β, and body mass index. A LOESS regression was used to model the association between age and BMI (left y‐axis), capturing the non‐linear trend across the age span and the association between age and Aβ (right y‐axis) (*n* = 245). Aβ is measured in CL. Aβ, amyloid beta; BMI, body mass index; CL, Centiloid.

Figure 2 shows the relation between PET Aβ and BMI. In models that included tau PET, there was no significant effect of tau or tau × time on change in BMI over time. In these models, a PET Aβ value of 18 Centiloids (i.e., threshold for Aβ positivity^18^) occurs at age 41 years, 2 years prior to the start of a decline in BMI (age 43 years). Following Aβ positivity, BMI was estimated to decrease by 0.6 kg/m^2^ by the time an individual reaches PET Aβ value of 60 Centiloids (which in prior studies was associated with having MCI^45^), and BMI is estimated to decrease by 1.2 kg/m^2^ by the time an individual reaches PET Aβ value of 100 (which in prior studies was associated with having AD clinical dementia^45^).

The model was re‐run including only the 191 participants who were cognitively stable at baseline to determine if associations remained prior to onset of clinical dementia. The negative association between time × PET Aβ remained significant (*β* = −0.013, *p *= 0.012).

### 3.4 BMI and cognitive functioning

**FIGURE 3 alz70387-fig-0003:**
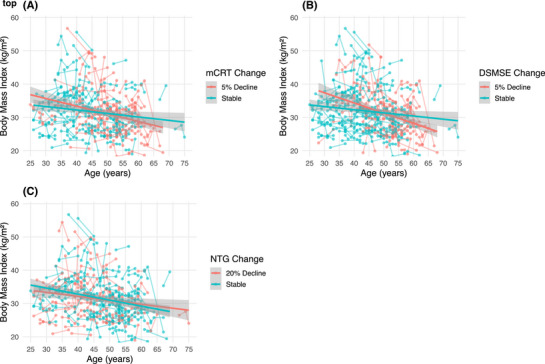
Associations between cognitive decline and body mass index. (A) Linear regression modeling of the association between age and BMI, comparing individuals who showed decline (orange line) versus those with a stable (blue line) performance on the mCRT (*n* = 431). (B) Linear regression modeling the association between age and BMI, comparing individuals who showed decline (orange line) versus those with a stable (blue line) performance on the DSMSE (*n* = 464) (C) Linear regression modeling of the association between age and BMI, comparing individuals who showed decline (orange line) versus those with a stable (blue line) performance on the between the NTG‐EDSD (*n* = 467). Visual models appear modest, but statistical models revealed significant associations between changes in BMI and both the mCRT and NTG‐EDSD. BMI, body mass index; DSMSE, Down Syndrome Mental Status Examination; mCRT, modified Cued Recall Test; NTG‐EDSD, National Task Group‐Early Detection Screen for Dementia.

Table 4 presents[Table alz70387-tbl-0003] the linear mixed model results for associations between cognitive performance and BMI across data collection cycles. In models including mCRT total score, time (*β* = −0.509, *p *= 0.004), age (*β* = −0.082, *p *= 0.034), biological sex (*β* = 2.184, *p *= 0.002), and the interaction of time × mCRT (*β* = 0.014, *p *= 0.011) were significant predictors of BMI. Across time, a positive association between mCRT and BMI emerges (i.e., a higher mCRT score is associated with higher BMI). Based on model estimates, a 5% decrease on the mCRT is associated with a 0.1 kg/m^2^ decrease in BMI. Figure 3 shows the association between mCRT across time and BMI. For models including DSMSE, time (*β* = −0.391, *p *= 0.03), age (*β* = −0.094, *p *= 0.010), and biological sex (*β* = 2.008, *p *= 0.002) were significant predictors of BMI. Similar to the model for mCRT, time and age had a negative effect on BMI. There was also a trend‐level interaction of time × DSMSE scores (*β* = 0.005, *p *= 0.076) on BMI; across time, a positive association between DSMSE scores and BMI was seen. Based on model estimates, a 5% decrease on the DSMSE was associated with a 0.3 kg/m^2^ decrease in BMI. Finally, in models including NTG‐EDSD, age (*β* = −0.121, *p *= 0.001), biological sex (*β* = 2.078, *p *= 0.002), and the interaction of time × NTG‐EDSD (*β* = −0.011, *p *= 0.039) were significant predictors of BMI. Both age and the interaction between time and NTG‐EDSD scores were negatively associated with BMI. This finidng means that as age and NTG‐EDSD scores increase, BMI decreases. Based on model estimates, a 21% increase on the NTG‐EDSD was associated with a 0.1 kg/m^2^ decrease in BMI.

**TABLE 4 alz70387-tbl-0004:** Linear mixed modeling for change in cognitive performance and body mass index.

		Estimates			
BMI	Fixed effects	*β*	SE β	*T*	*p*‐value
mCRT	Intercept	31.866	2.598	12.266	< 0.001^***^
	Time	−0.509	0.175	−2.906	0.004^**^
	Age	−0.082	0.038	−2.134	0.034^*^
	Biological sex	2.184	0.683	3.199	0.002^**^
	Premorbid intellectual disability	−0.391	0.556	−0.704	0.482
	*APOE* ε4	0.159	0.734	0.217	0.829
	mCRT	0.030	0.026	1.120	0.263
	Time × mCRT	0.014	0.006	2.545	0.011^*^
DSMSE	Intercept	32.324	2.700	11.991	< 0.001^***^
	Time	−0.391	0.180	−2.167	0.031^*^
	Age	−0.094	0.036	−2.596	0.010^*^
	Biological sex	2.008	0.650	3.088	0.002^**^
	Premorbid intellectual disability	−0.541	0.546	−0.992	0.322
	*APOE* ε4	0.131	0.681	0.192	0.848
	DSMSE	0.019	0.016	1.175	0.240
	Time × DSMSE	0.005	0.003	1.780	0.076
NTG‐EDSD	Intercept	34.873	2.079	16.772	< 0.001^***^
	Time	−0.048	0.056	−0.859	0.391
	Age	−0.121	0.035	−3.454	0.001^**^
	Biological sex	2.078	0.650	3.197	0.002^**^
	Premorbid intellectual disability	−0.802	0.513	−1.562	0.119
	*APOE* ε4	0.095	0.685	0.139	0.890
	NTG‐EDSD	0.021	0.020	1.043	0.295
	Time × NTG‐EDSE	−0.011	0.005	−2.070	0.039^*^

*Note*. *N *= 431 (mCRT), 464 (DSMSE), and 467 (NTG‐EDSD). Unstandardized coefficients (*β*s) are presented. All models include a random intercept and site was included as a random intercept.

Abbreviations: *APOE*, apolipoprotein E; BMI, body mass index; DSMSE, Down Syndrome Mental Status Examination; mCRT,  modified Cued Recall Test; NTG‐EDSD, National Task Group‐Early Detection Screen for Dementia.

* *p* < 0.05.

** *p* < 0.01.

*** *p* < 0.001.

Models were re‐run to include only the 342 participants deemed to be cognitively stable at baseline. There continued to be a positive time × mCRT interaction on BMI change (*β* = 0.016, *p *= 0.021). There also continued to be a trend‐level time × DSMSE effect on BMI change (*β* = 0.006, *p *= 0.078). However, there was no longer a significant time × NTG‐EDSD effect on BMI change (*β* = −0.011, *p *= 0.178), potentially reflecting that the NTG‐EDSD is dependent on observed changes reported by study partners rather than direct measures of cognitive functioning.[Fig alz70387-fig-0002]

### 3.5 Baseline BMI status and cognitive decline

Follow‐up analyses were conducted to understand differences in BMI decline in relation to cognitive decline by baseline BMI status (normal/underweight, overweight, and obese). Compared to participants who were normal or underweight at baseline, those who were obese had a flatter decline in BMI in relationship to decline in mCRT score (*β* = 0.144, *p* = 0.002). There was not a significant difference in the association between mCRT decline and BMI decline between participants who were overweight and those who were normal or underweight at baseline (*β* = 0.047, *p* = 0.337). Similarly, on the DSMSE, participants who were obese at baseline showed a flatter decline in BMI in relation to decline in DSMSE score (*β* = 0.086, *p* < 0.001) than those who were underweight or normal weight at baseline. There was no statistically significant difference in the association between DSMSE decline and BMI decline in participants who were overweight versus underweight or normal weight (*β* = 0.033, *p* = 0.198). There was not an association between decline in NTG‐EDSE and BMI decline by baseline BMI status (Obese: *β* = −0.011, *p* = 0.749; Overweight: *β* = −0.013, *p* = 0.719). Figure S1 (mCRT) and Figure S2 (DSMSE) depict significant findings.[Table alz70387-tbl-0004], [Fig alz70387-fig-0003]

## 4 DISCUSSION

In the neurotypical population, unintentional weight loss occurs in the years preceding the onset of AD dementia.^2^, ^4^ However, little is known about the relationship between weight loss and the onset of AD dementia in DS. Results from the current longitudinal study built on prior cross‐sectional research^27^, ^28^, ^29^ that suggested that the onset of decreasing BMI occurs around the time that early AD pathology (i.e., amyloid burden) begins in adults with DS. Findings from the present study show that on average, adults with DS show declines in BMI beginning in their early 40s. This finding is consistent with previous research also showing that adults with DS experience BMI declines after the third decade of life.^25^ Based on our model estimates, by age 53 years, an adult with DS would be expected to experience a 1.5 kg/m^2^ decrease in their BMI relative to their BMI at age 42 years. The effect continues with age so that, compared with their BMI at 42, at age 64 years, an adult with DS would be expected to experience a 3.0 kg/m^2^ decrease in BMI. It is important to note that the age of onset of initial decline in BMI (43 years) in the current study is slightly older than was reported in our prior cross‐sectional study.^28^ This discrepancy may occur because longitudinal data provide a more precise estimate of within‐person BMI decline. Premorbid ID^46^, ^47^ level and *APOE* ε4 status^28^ were not associated with changes in BMI over time. These findings are consistent with previous research in both the general population^48^ and individuals with DS.^28^

In the current study, a higher baseline PET Aβ level was significantly associated with greater decline in BMI over time in adults with DS. Indeed, the BMI of adults with DS was estimated to decrease by 0.6 kg/m^2^ from the time of Aβ positivity (18 centiloids (CL))^18^ to having a PET Aβ value of 60 CL and by 1.2 kg/m^2^ at a PET Aβ value of 100 CL. The association between higher baseline Aβ and lower BMI over time held true even when only examining the subset (*n* = 191) of adults with DS who were cognitively stable and in models controlling for age. Thus, the association between Aβ burden and BMI decline occurs prior to clinical AD dementia and does not appear to be attributable only to age‐related effects on BMI. The potential biological mechanisms linking weight loss to early Aβ burden are not fully understood. However, in mouse models, Aβ deposition disrupts hormone regulation, including leptin and adiponectin signaling,^10^, ^11^ and is posited to affect brain regions related to appetite control and energy regulation, such as the entorhinal cortex and hippocampus.^12^, ^13^ In our study, there was no statistical association between baseline tau PET and BMI decline. The lack of significant findings may be due to the smaller sample of participants with available tau data (relative to Aβ data), which limited statistical power. In addition, an effect of tau burden on BMI decline may have been overshadowed by stronger associations with Aβ deposition. Given the short time lag between Aβ positivity and tau positivity in adults with DS compared to LOAD,^45^, ^49^ it may be challenging to disentangle the individual contributions of tau relative to Aβ pathology. Alternatively, it is possible that BMI decline is more closely linked to biological mechanisms triggered by Aβ than tau burden.

Decline in memory and cognitive functioning across time was also associated with BMI decline in adults with DS, even in models controlling for age and other sociodemographic characteristics. For example, a 5% decrease in the mCRT Total score was associated with a 0.1 kg/m^2^ decrease in BMI. Similarly, a 21% increase on the NTG‐EDSD corresponded to a 0.1 kg/m^2^ decrease in BMI. Results held true when only examining participants deemed to be cognitively stable, suggesting that BMI decline coincides with early and subtle AD symptomatology. Moreover, in models controlling for age, adults with DS who were obese at baseline had a flatter decline in cognition (mCRT and DSMSE) across time than those who started with a lower BMI (underweight or normal weight). This finding may similarly reflect that those with lower BMIs were farther along in the unfolding of AD symptomatology than those with higher BMIs. Together, these findings suggest that BMI decline may be part of early AD expression and observed years prior to dementia onset. However, it is important to note that in later disease stages (e.g., following clinical AD dementia onset), cognitive decline and other AD symptoms may exacerbate weight loss in adults with DS. Indeed, outside of DS, AD‐related impairments in memory and attention, associated with medial temporal lobe reduction,^50^ may contribute to forgetting to eat, and other dementia symptoms such as difficulty swallowing may result in reduced caloric intake.^51^

The present study had both strengths and limitations. In terms of strengths, the study leveraged up to four cycles of data (each spaced ≈16 months apart) in a large cohort of adults with DS, most of whom did not yet have clinical AD dementia. The study used PET imaging to assess AD pathology with methods harmonized across sites. Cognitive functioning was assessed using a battery of directly administered and informant‐based measures. In terms of limitations, the use of BMI, while practical, does not indicate the specific nature of weight loss (e.g., whether it stems from reductions in muscle mass, bone density, or fat mass). Incorporating body composition scanners into future studies would better identify the sources of weight loss and elucidate the underlying mechanisms. This study cannot fully determine if weight loss was intentional or unintentional. None of the participants had weight loss surgery or were reported to be taking glucagon‐like peptide‐1 weight loss medications. Many participants had hypothyroidism (60%) and obstructive sleep apnea (36%). These conditions were typically longstanding, but it is possible that their severity altered during the course of the study. In addition, the main results were unaltered in follow‐up analyses when participants with the greatest weight loss (>10 units, *N* = 10 [2.14%]) were removed. However, separating intentional from unintentional weight loss will be important in future studies. The present study also lacked diversity, with 92% of participants identifying as White, non‐Hispanic. To enhance the generalizability of findings, future research should include samples with broader demographic variability, including measures of economic disadvantage as well as race/ethnicity and biological sex.

In summary, weight loss could be an important sign of impending AD dementia in adults with DS. Further research is also need to disentangle the time‐ordered nature of BMI change, age, Aβ deposition, and cognitive decline in DS in order to identify the biological mechanisms that may drive associations. In the meantime, the findings have important clinical implications. Weight change should be included in DSAD screenings and weight should be monitored in annual visits of adults with DS.

## CONFLICT OF INTEREST STATEMENT

The authors declare no conflicts of interest. Author disclosures are available in the Supporting Information.

## CONSENT STATEMENT

Informed consent and/or assent were obtained for all participants.

## Supporting information



Supporting Information

Supporting Information
