# Supplementary material for: Longitudinal study of body mass index in relation to Alzheimer's disease pathology and symptomatology in Down syndrome
Source: Alzheimers Dement. 2025 Jun 22;21(6):e70387. doi: 10.1002/alz.70387 (PMC12183108; doi:10.1002/alz.70387)
Supplement: Supplementary file 2 — Supporting Information [file ALZ-21-e70387-s001.docx]

**
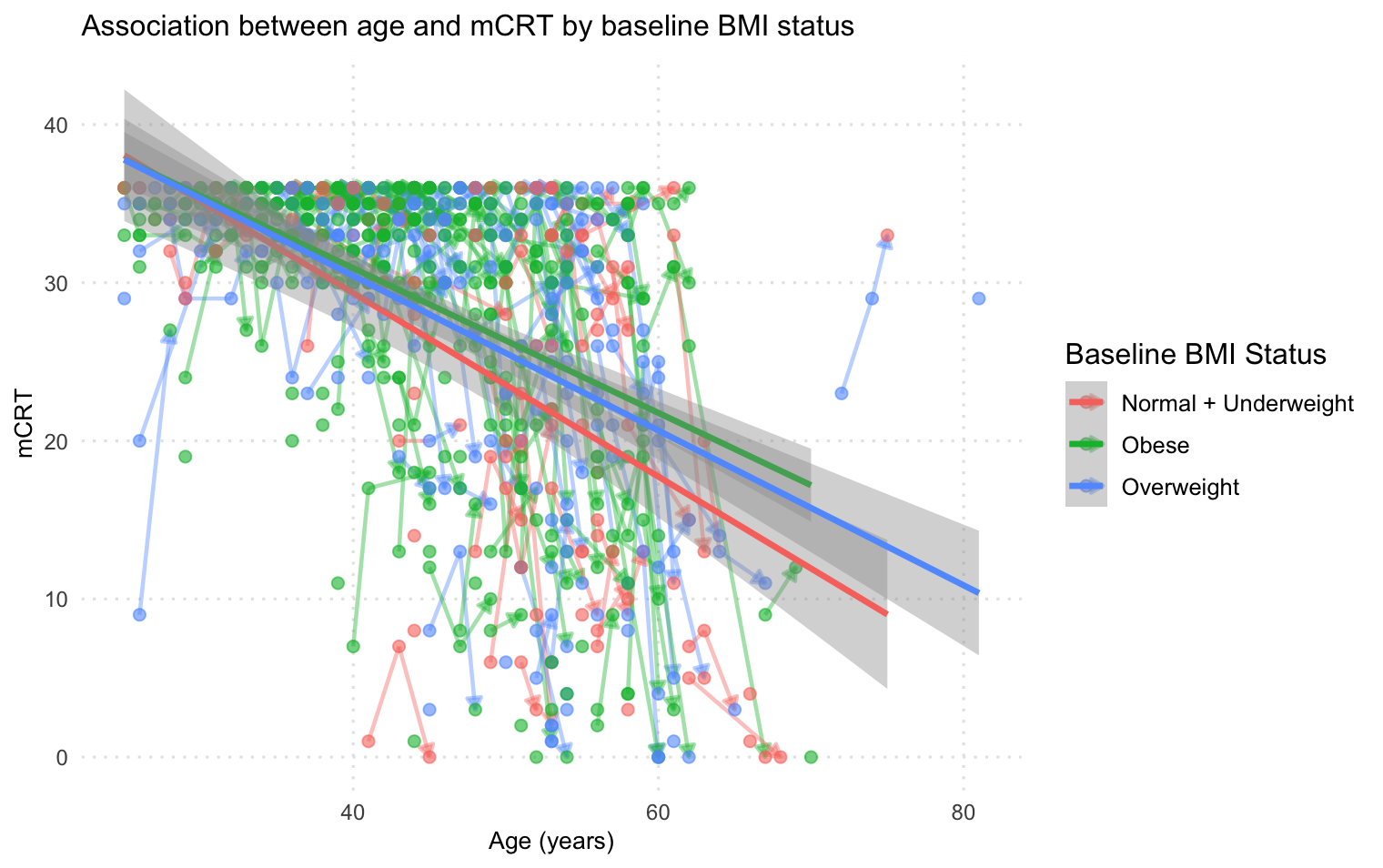
**

**Supplemental Figure 1. Associations between mCRT and age by baseline BMI status**. Linear regression modeling the association between age and modified Cued Recall (mCRT), compared by baseline body mass index (BMI) status.


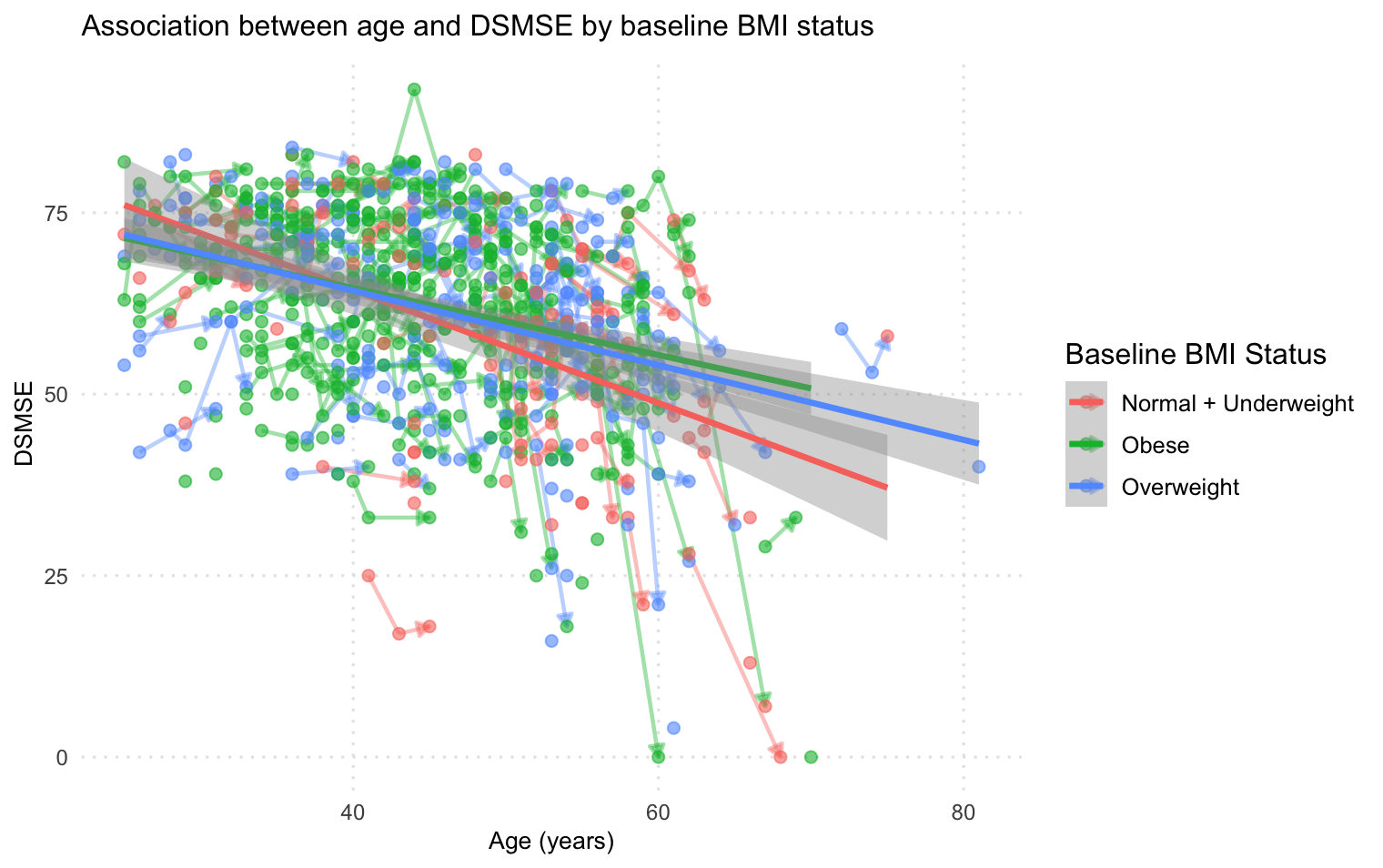


**Supplemental Figure 2. Associations between DSMSE and age by baseline BMI status**. Linear regression modeling the association between age and Down Syndrome Mental Status Examincation (DSMSE) compared by baseline body mass index (BMI) status.
